# Another Raid on the Civil Hospitals

**Published:** 1915-10-16

**Authors:** 


### ANOTHER RAID ON THE CIVIL HOSPITALS.

The title placed at the head of this article does not refer to any operations or enterprises of the enemy. The reference is rather to those members of the national household who are grouped compendiously under the name and dignity of the War Office. These exalted authorities, to put the matter quite plainly, have issued a request for an increased number of beds to be allocated to them in the civil hospitals, and for the reception of sick and wounded soldiers. It is, we hope, not necessary for us to repeat that we are most anxious that our brave fellows shall have every succour which medical science and skilled nursing can afford them. We quite agree, too, that theirs is the first claim, and that if unrelieved suffering must exist it must exist among the civil population rather than among the soldiers. Hence, if there is no other way to obtain adequate treatment for the latter the civil hospitals must provide it, even though the original purpose of these hospitals—the medical and surgical care of the sick and suffering poor—be sacrificed in the process. But before this sacrifice is made there ought to be submitted plain evidence of its necessity.

The method of procedure which is being adopted is, we believe, highly unfair to the civil hospitals and to the populations which depend on them. First, the War Office asks for beds, and managers readily, perhaps too readily, grant them. Then after some months the request is repeated; and there is every reason to anticipate that ere long it will be urged once more. It is difficult, we recognise, for managers to resist what seems to be a national claim, and hence there is the danger that by a graduated series of steps the rights of the civil population will be sacrificed. If the War Office is at the end of its hospital resources its duty is to commandeer the civil hospitals in so far as this action is necessary. In this case the responsibility would be openly accepted by those who ought to bear it, and evidence will have to be produced to justify the decision. But the present gradual invasion places responsibility on the hospital managers, who have either to refuse what appears as a patriotic claim or to prejudice the civilians who are their peculiar care. Before they can defend this latter decision they ought to know on the evidence of facts and figures that no other course is available.

The strength of this position is increased when it is remembered that already the civil hospitals have yielded much. Concerning the actual situation we have no desire to quarrel with what has been done, but we certainly suggest that the time has come when no more should be yielded except upon specific proof of an emergency for which no other relief is possible. If this evidence is forthcoming, well and good. But until it appears it seems to us to be the duty of managers to state plainly that their resources cannot be further invaded without the infliction of suffering and loss on the civil population, and that for such a position the War Office must openly accept the full responsibility. For our own part we do not believe that it is impossible to organise and equip further military hospitals, and nothing but actual evidence will make us depart from this view. By all means give the soldiers the best we can command, but in doing this let us not, without overwhelming necessity, inflict injustice on other parts of the nation.

There is still another method by which the civil hospitals may be relieved. It is that the War Office should arrange greater convalescent facilities for soldiers who no longer need hospital treatment. On every hand we hear of wards occupied by soldiers the greater part of whom are quite ready to be discharged and have need of nothing but good food, rest, and fresh air. Nothing but organisation is required to secure this end and to release the hospitals for their proper work. When the War Office is able to show that it is utilising to the full the opportunities placed at its disposal, and that apart from the civil hospitals it has no remaining resource, the time will have arrived to listen to its further requests.

